# Supplementary figures and images for: Modes of Interaction between Individuals Dominate the Topologies of Real World Networks
Source: PLoS One. 2015 Mar 20;10(3):e0121248. doi: 10.1371/journal.pone.0121248 (PMC4368763; doi:10.1371/journal.pone.0121248)

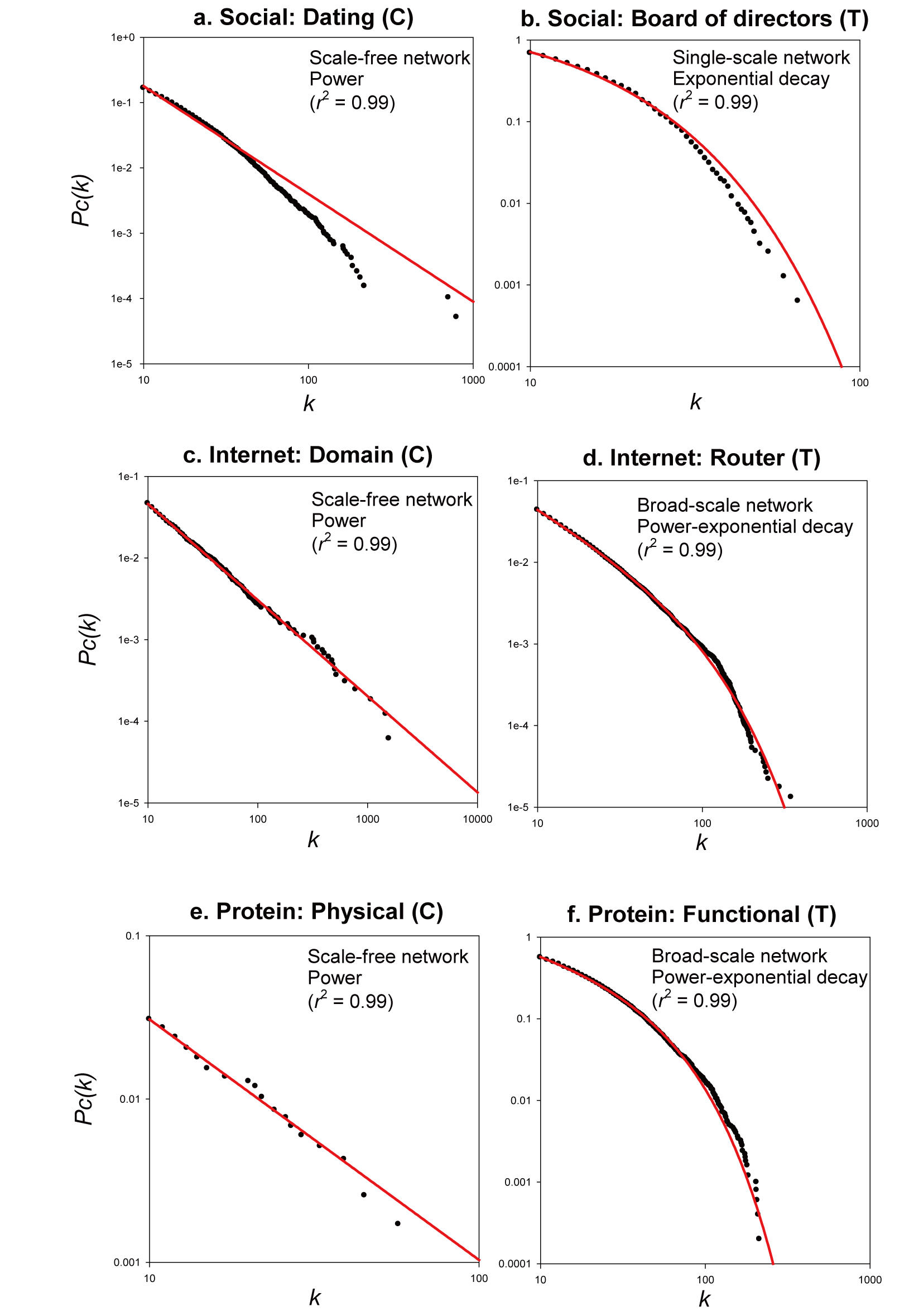

Supplement: S1 Fig — Three contact-centric (indicated by the letter “C”) networks and three task-centric (indicated by the letter “T”) networks were analyzed, highlighting the degree distribution tails. (TIF) [file pone.0121248.s001.tif]

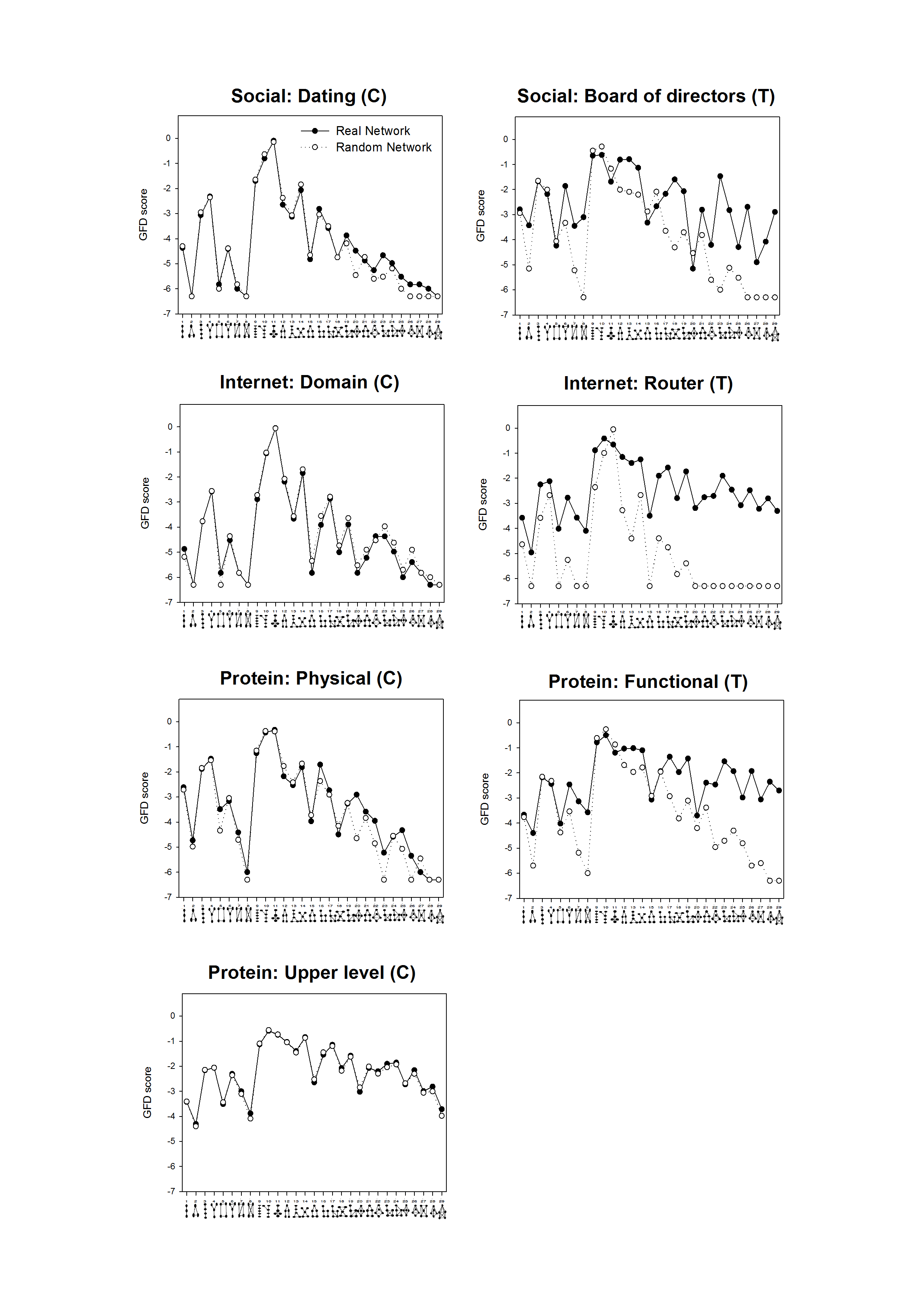

Supplement: S2 Fig — Four contact-centric (indicated by the letter “C”) networks and three task-centric (indicated by the letter “T”) networks and their randomized networks were analyzed. (TIF) [file pone.0121248.s002.TIF]

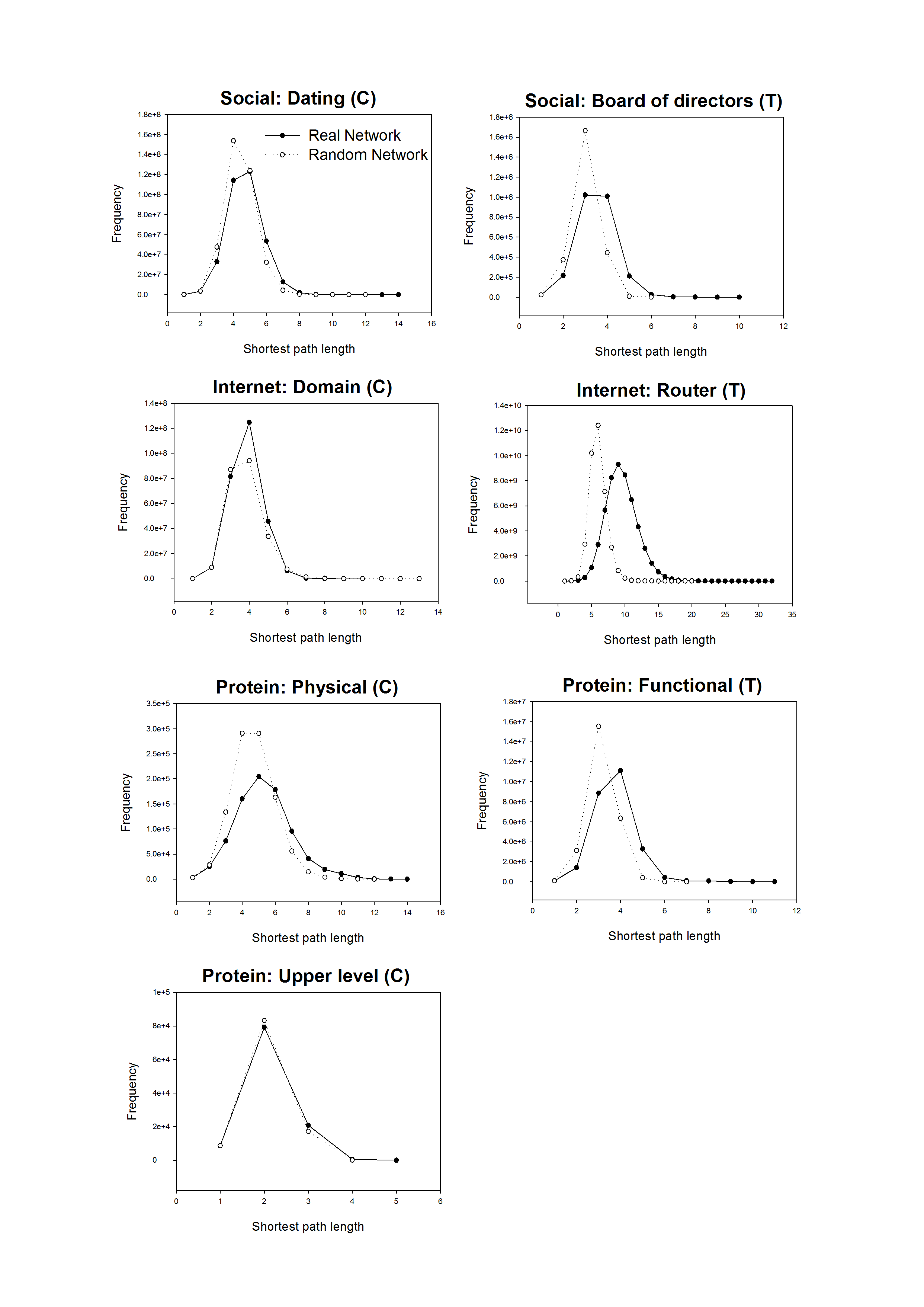

Supplement: S3 Fig — Four contact-centric (indicated by the letter “C”) networks and three task-centric (indicated by the letter “T”) networks and their randomized networks were analyzed. (TIF) [file pone.0121248.s003.tif]

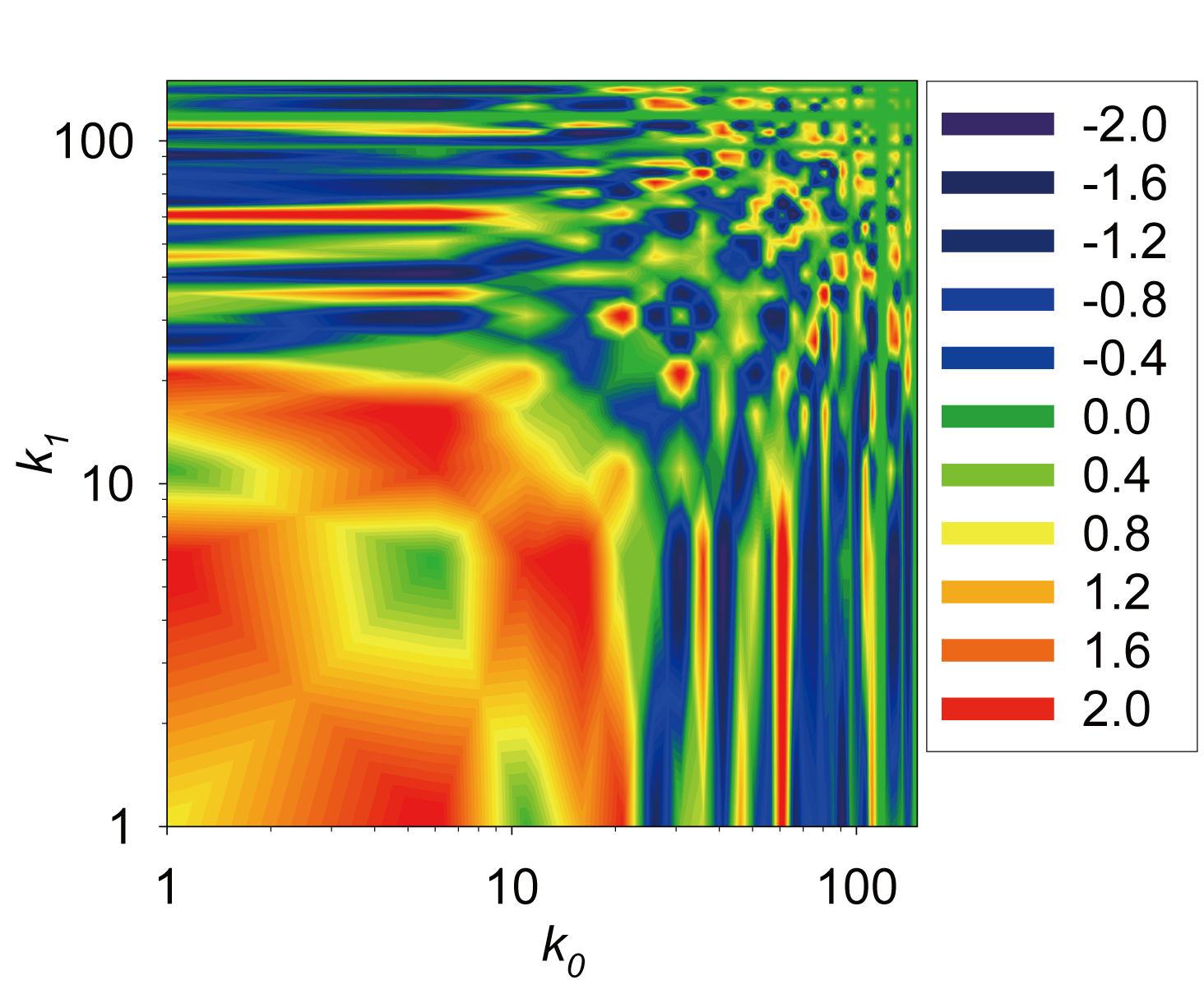

Supplement: S4 Fig — (TIF) [file pone.0121248.s004.tif]

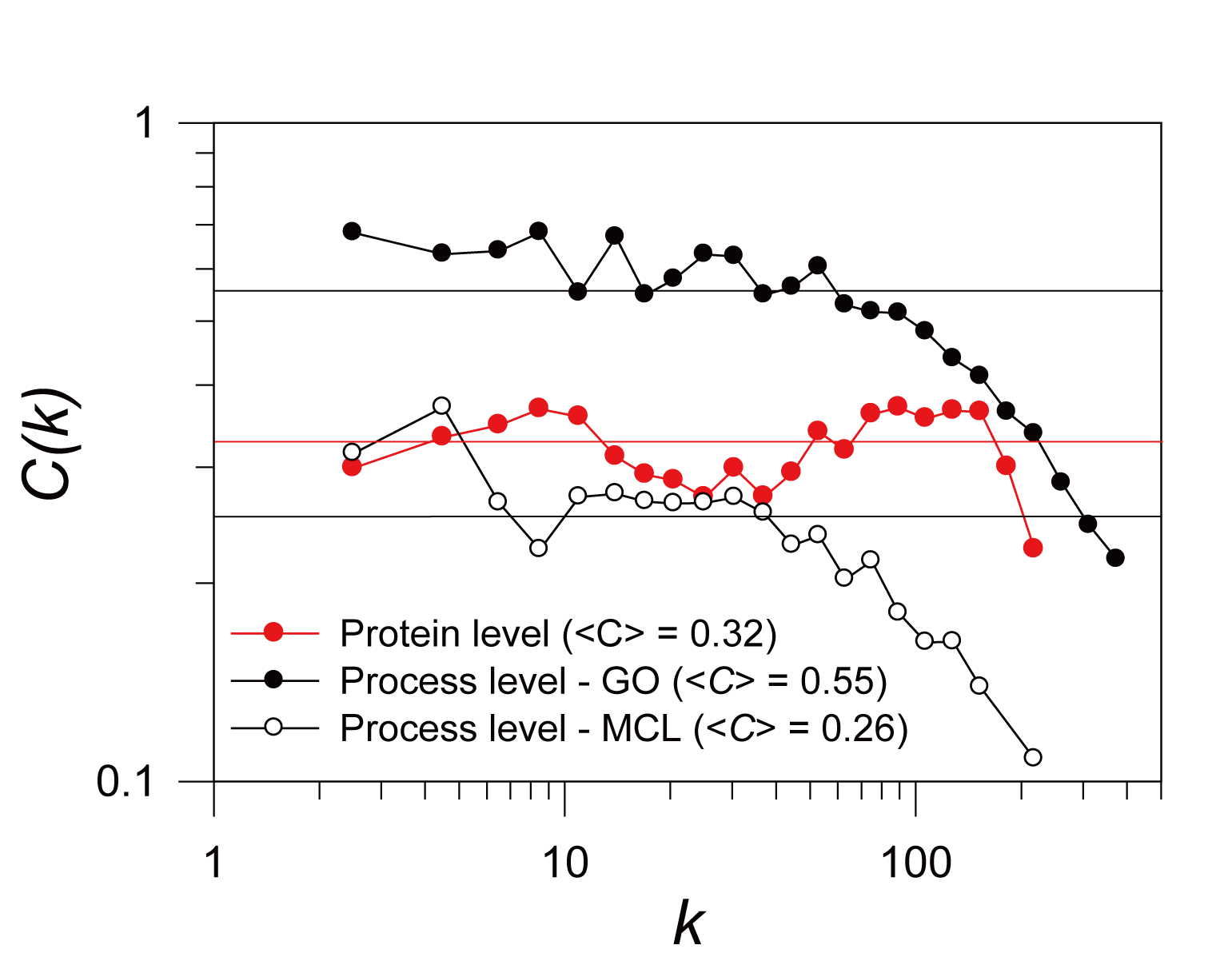

Supplement: S5 Fig — Both a network of previously defined Gene Ontology biological processes (in which edges are defined between 7th level GO-BP annotations based on sharing of annotated proteins; labeled “Process level – GO” in the plot) and the network of biological processes inferred by MCL clustering of a functional protein network (“Process level – MCL”) show hierarchical organization, while the functional protein network itself (“YeastNet core”) shows a nonhierarchical organization, in which clustering coefficients for a given degree connectivity (C(k)) trend near the average clustering coefficient for the entire network (). (TIF) [file pone.0121248.s005.tif]
