# Supplementary material for: Modes of Interaction between Individuals Dominate the Topologies of Real World Networks
Source: PLoS One. 2015 Mar 20;10(3):e0121248. doi: 10.1371/journal.pone.0121248 (PMC4368763; doi:10.1371/journal.pone.0121248)
Supplement: S1 Table — (PDF) [file pone.0121248.s006.pdf]

S1 Table. Summary of the networks analyzed in this study.

**Network**	**Edge representation**	**Node representation**	**# node**	**# edge**	**<*k*>**
Protein physical interactions (CCSB-YI1)[1]	Contact-centric	Protein	1,173	1,547	2.6
Protein functional interactions (YeastNet core)[2]	Task-centric	Protein	5,053	47,000	18.6
Protein processes[2]	Contact-centric	Biological process	331	4,348	26.3
Dating[3]	Contact-centric	Person	19,481	58,978	6.1
Board of directorships[4]	Task-centric	Person	1,586	11,540	14.6
Internet routers[5]	Task-centric	Router	228,298	320,168	2.8
Internet domains[5]	Contact-centric	Domain	16,413	31,031	3.8

[Table references]

1. Yu H, Braun P, Yildirim MA, Lemmens I, Venkatesan K, et al. (2008) High-quality binary protein interaction map of the yeast interactome network. Science 322: 104-110.

2. Lee I, Li Z, Marcotte EM (2007) An improved, bias-reduced probabilistic functional gene network of baker's yeast, Saccharomyces cerevisiae. PLoS ONE 2: e988.

3. Holme P, Edling CR, Liljeros F (2004) Structure and time evolution of an Internet dating community. Social Networks 26: 155-174.

4. Davis GF, Yoo M, Baker WE (2003) The small world of the American corporate elite, 1982-2001. STRATEGIC ORGANIZATION 1: 301-326.

5. Vazquez A, Pastor-Satorras R, Vespignani A (2002) Internet topology at the router and autonomous system level. arXiv:cond-mat/0206084.
